# Hepatocellular carcinoma among patients with chronic hepatitis B in the indeterminate phase

**DOI:** 10.1111/jvh.13914

**Published:** 2024-05-08

**Authors:** Lung‐Yi Mak, Leland J. Yee, Robert J. Wong, Christian B. Ramers, Catherine Frenette, Yao‐Chun Hsu

**Affiliations:** ^1^ Department of Medicine, Queen Mary Hospital The University of Hong Kong Hong Kong Special Administrative Region China; ^2^ Gilead Sciences Foster City California USA; ^3^ Division of Gastroenterology and Hepatology Stanford University School of Medicine Palo Alto California USA; ^4^ Division of Gastroenterology and Hepatology Veterans Affairs Palo Alto Health Care System Palo Alto California USA; ^5^ Laura Rodriguez Research Institute Family Health Centers of San Diego San Diego California USA; ^6^ University of California San Diego School of Medicine La Jolla California USA; ^7^ Division of Gastroenterology and Hepatology E‐Da Hospital Kaohsiung Taiwan; ^8^ School of Medicine, College of Medicine I‐Shou University Kaohsiung Taiwan; ^9^ Institute of Biomedical Informatics National Yang Ming Chiao Tung University Taipei Taiwan; ^10^ Division of Gastroenterology and Hepatology Fu‐Jen Catholic University Hospital New Taipei Taiwan

**Keywords:** grey zone, indeterminate, low‐level viremia, therapy

## Abstract

Hepatitis B virus (HBV) infection is a dynamic disease where patients progress through several stages defined by HBV e‐antigen (HBeAg) status, HBV‐DNA levels and transaminase elevations, with antiviral therapy indicated only in specific stages. However, some patients cannot be classified into one of the stages and are said to fall into an ‘indeterminate phase’ or ‘grey zone’. Exact definitions of the indeterminate phase vary from guideline to guideline as a result of different cut‐off values for biomarker measurements. Data suggest that as many as 50% of HBV patients may be in an indeterminate phase and may not rapidly transition out of this phase. Clinical data that suggest these patients are at increased risk of hepatocellular carcinoma (HCC) are complemented by molecular evidence of integrations of HBV‐DNA into the host genome, chromosomal translocations and immune activation despite liver enzymes that may suggest lack of inflammation. Antiviral therapy reduces these hepatocarcinogenic mechanisms and is reflected in a reduction of fibrosis and HCC risk. We review key data on patients in the indeterminate phase, with emphasis on HCC as an outcome. We take a holistic approach and link new biological data with clinical observations as well as examine the potential role of antiviral therapy in reducing HCC risk among patients in the indeterminate phase. With the availability of safe and effective oral antivirals, consideration must be given as to how much residual risk of HCC should be tolerated among patients in the indeterminate phase.

**Abbreviations**
95% CI: 95% confidence interval
ALT: alanine amino transferase
HR: hazard ratio
RR: relative risk

## 1 INTRODUCTION: HEPATITIS B AND INDETERMINATE PATIENTS

The public health impact of hepatitis B Virus (HBV) infection remains high, while overall linkage of patients to care remains low.^1^ Among the estimated 258 million hepatitis B surface antigen (HBsAg)‐positive individuals in 2022, only 36 million are diagnosed.^2^ Of the 83.3 million estimated to be treatment‐eligible based on 2022 criteria, only 6.8 million are actually treated.^2^ These data are concerning, as without treatment, individuals with chronic HBV have an estimated 30%–40% lifetime risk of developing cirrhosis or hepatocellular carcinoma (HCC).^3^ HBV accounts for over 850,000 deaths annually,^2^ with an estimated two people dying each minute.^4^ While treatment of HBV with nucleos(t)ide analogues (NAs) are not curative, current first‐line regimens are well‐tolerated and effective in suppressing viral replication with little to no detectable resistance.^5^ Importantly, treatment results in a significant reduction in the risk of liver‐related complications and HCC.^6^

**FIGURE 1:**
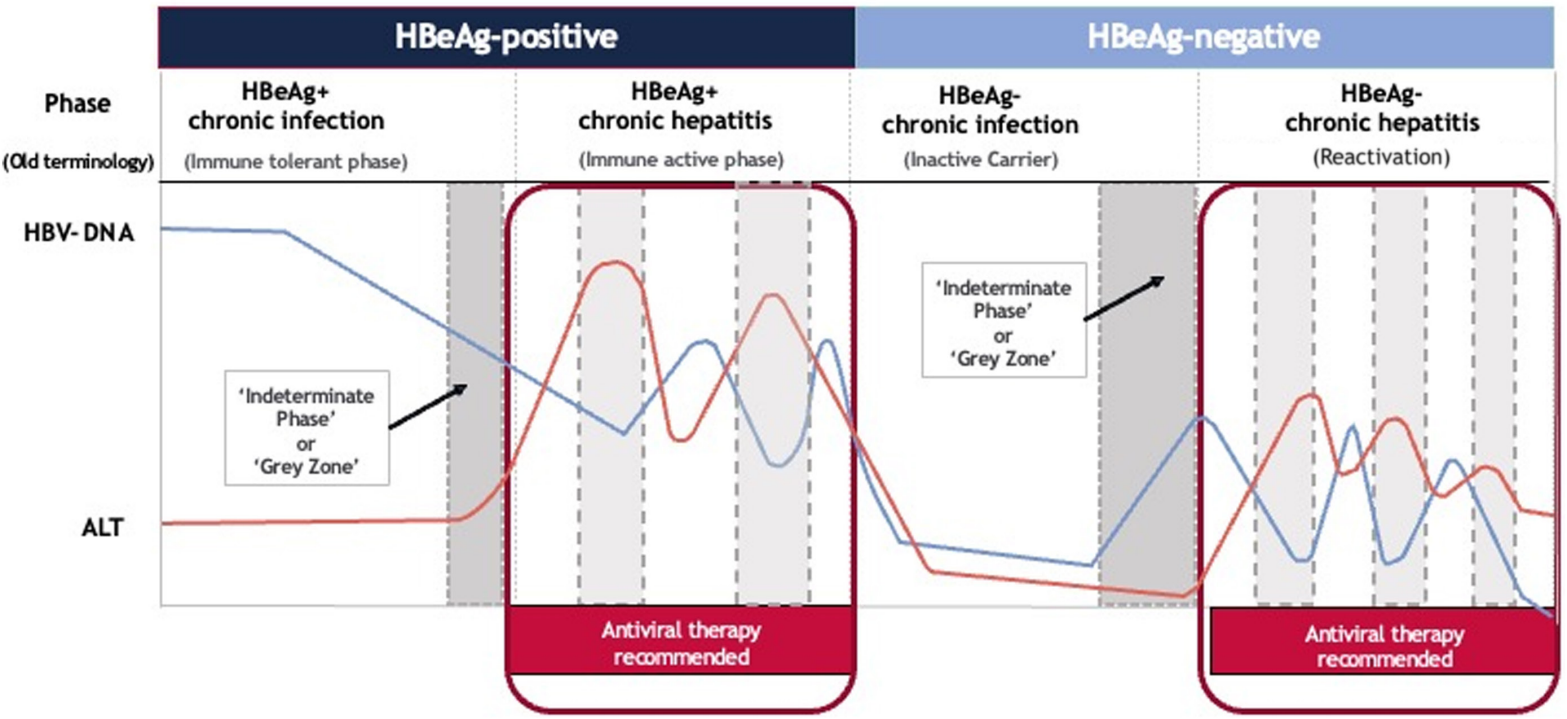
Simplified cartoon diagram of the stages of hepatitis B natural history. Examples of indeterminate phases that do not fit into any disease stages are highlighted in grey. The low to moderately elevated HBV‐DNA and high ALT and stages are highlighted in light grey (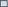). In this phase, additional factors that may lead to ALT elevations such as steatosis or alcohol use, should be considered. The high HBV‐DNA and low to moderately elevated ALT phase is depicted in dark grey (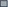) and is the focus of the present review. Figure not to scale.

**FIGURE 2:**
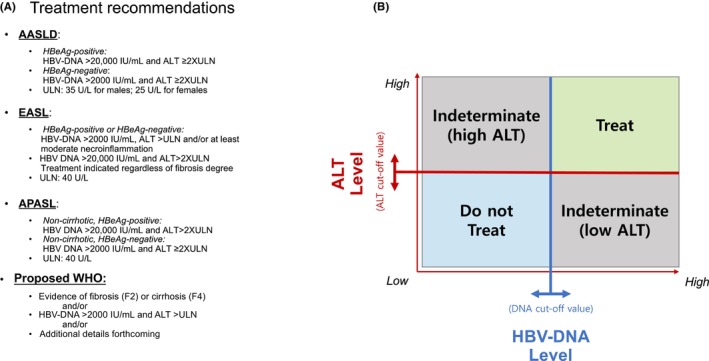
Schematic diagram of major guidelines and the indeterminate phase. (A) presents the HBV‐DNA and ALT thresholds for treatment by society guideline (AASLD,^39^ EASL,^40^ APASL,^38^ and WHO^7^), along with the different definitions for the upper limit of normal (ULN) of ALT. The proposed WHO guidelines should present a more simplified set of treatment criteria that eliminates the indeterminate‐low or moderate ALT phase (HBV‐DNA >2000 IU/mL and low or moderately elevated ALT). (B) depicts the different scenarios where treatment is indicated and indeterminate. The relative size of each of the patient segments varies by guidelines, as different HBV‐DNA thresholds will shift the horizontal line ( 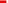 ) up or down, while different ALT values ( 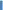 ) will shift the vertical line left or right. For patients in indeterminate–high ALT category (upper left box), comorbid conditions such as steatosis and alcohol use may be present, and they should be evaluated accordingly. Patients in the indeterminate‐low ALT category (lower right box) reside in a phase where hepatocarcinogenic mechanisms are under way as evidenced by data from clinical and molecular studies.

HBV infection has historically been viewed as a dynamic disease, progressing through various stages primarily delineated by hepatitis B e‐antigen (HBeAg) status, serum levels of HBV‐DNA and serum levels of alanine aminotransferase (ALT) as a surrogate marker of hepatic necroinflammation (Figure 1). In the absence of significant histological disease or family history of HCC, most historical practice guidelines recommend treatment when both HBV‐DNA and ALT levels are elevated (Figure 1), namely the immune active and reactivation phases. With guidelines utilizing different thresholds for HBV‐DNA and ALT for the initiation of therapy, and differences in the definition of the upper limit of normal (ULN) for ALT (Figure 2), there is the potential for confusion given variations in treatment eligibility criteria across society recommendations and the complexity has contributed to gaps in treatment due to lack of clarity about who should or should not be treated. The proposed updates to the World Health Organization (WHO) guidelines  aim to expand recommended treatment criteria, perhaps an acknowledgement of a changing risk/benefit landscape as evidence emerges regarding both the dynamic nature of HBV, and the variable long‐term outcomes of patients in indeterminate phases, where treatment has heretofore not been recommended.^7^

Clinically, HBV infection does not always neatly fit into the currently‐defined stages of HBV natural history, with some patients unable to clearly fulfill treatment criteria. For example, patients may exhibit HBV‐DNA >2000 IU/mL yet have ‘normal’ ALT levels, or conversely, present with HBV‐DNA ≤2000 IU/mL but elevated ALT levels, where possible comorbidities such as steatosis or alcohol use warrant additional evaluation. Patients in either of these categories who refuse diagnostic liver biopsy to assess the degree of fibrosis, are considered to be in an ‘indeterminate phase’ or ‘grey zone’ (Figures 1 and 2),^8^ with the exact definition of who falls into this category varying from guideline to guideline due to variations in HBV‐DNA and ALT cut‐offs (Figure 2). Patients may fall into an ‘indeterminate phase’ in both the broad HBeAg‐positive (HBeAg+) or HBeAg‐negative (HBeAg−) categories. Recent studies have suggested that the number of patients in the indeterminate phase may be as high as 30%–50%.^9^, ^10^, ^11^, ^12^, ^13^ While many use the terms ‘indeterminate phase’ or ‘grey zone’ interchangeably, we will use the term ‘indeterminate phase’ for consistency.

Recent analyses suggest that patients in the indeterminate phase may continue to be at risk for fibrosis progression, HCC development, and liver‐related complications.^9^, ^11^, ^14^, ^15^, ^16^ Increased understanding of HBV virology in recent years has illuminated potential pathogenic mechanisms that may contribute to increased risk profiles. This review summarizes current data on the risk of HCC among HBV patients in the indeterminate phase and the potential benefit of NA therapy. For conciseness, this review focuses on patients in the HBeAg− indeterminate phase, in particular viremic patients without ‘high’ ALT, as patients with viral levels <2000 IU/mL and elevated ALT may have other underlying comorbidities outside the scope of this review.

## 2 HBV AND HEPATOCARCINOGENESIS

### 2.1 HBV‐DNA levels and HCC risk

The Risk Evaluation of Viral Load Elevation Liver Disease/Cancer‐Hepatitis B Virus (REVEAL‐HBV) Study, a community‐based study of HBV natural history conducted in seven townships in Taiwan that involved 23,820 untreated patients (85% HBeAg−), has provided considerable insights on the relationship between HBV‐DNA levels and clinical outcomes.^17^ In a landmark paper, Chen and colleagues showed that increasing HBV‐DNA viral levels are associated with increasing HCC risk across a range of viral titres.^18^ Similar relationships between increasing viral levels and increased risk of other liver‐related outcomes such as cirrhosis and liver‐related mortality have also been reported.^19^, ^20^, ^21^ Interestingly, the relationship of HBV‐DNA levels and the risk of HCC was observed for both HBeAg+ and HBeAg− participants, with the strongest association among non‐cirrhotic HBeAg− participants with normal ALT levels.^17^, ^18^

An important finding from REVEAL was lower HCC risk for individuals with HBV‐DNA <2000 IU/mL compared to those with higher levels. HBV‐DNA ≥2000 IU/mL was associated with a higher risk of disease progression or HCC, regardless of HBeAg status or ALT level.^18^ It is important to note that the REVEAL study employed an earlier generation of HBV‐DNA assay (measured in copies/mL), where 10,000 copies/mL approximates 2000 IU/mL with second generation assays (1 IU/mL approximates 5 copies/mL).^22^ With a lower limit of detection of 300 copies/mL and an upper limit of 1 million copies/mL, earlier assays also had a much more limited range of detection.^18^ While the relationship between viral levels and HCC is firmly established at lower levels of HBV‐DNA, recent studies using newer assays suggest that the relationship between HCC risk and very high viral levels (>10^6^ IU/mL) may be more nuanced.^23^, ^24^

Hazard ratios (HRs) for HCC development in viral categories >10,000 copies/mL (~2000 IU/mL) were higher than for <10,000 copies/mL.^18^ Additional studies conducted in different populations outside of REVEAL have confirmed the observations that HBV‐DNA levels >2000 IU/mL are associated with increased risk of negative outcomes like cirrhosis and HCC.^15^, ^19^, ^25^, ^26^ Accordingly, the lower cut‐off of 2000 IU/mL has been incorporated into clinical practice. Nevertheless, HCC risk persists among individuals with lower levels of HBV‐DNA, albeit at a lower level compared to those with DNA levels >2000 IU/mL. In REVEAL, even those with undetectable DNA (<300 copies/mL) had an HCC incidence of 108/100,000 person‐years, underscoring the point that while HCC risk is reduced in the lower HBV‐DNA tiers,^18^ it is nevertheless present. Given the association of HBV‐DNA levels with HCC risk and the availability of NAs that are safe and highly effective in HBV‐DNA suppression, it can be argued that therapeutic efforts to maximize risk reduction would directly lead to individual and public health benefits.

### 2.2 ALT as a marker of hepatic inflammation

While ALT is often used as a surrogate marker of hepatic inflammation, its incorporation into treatment criteria is largely historical. The ALT 2× ULN cut‐off was originally used as entry criterion for studies of interferon in HBV treatment, without conclusive evidence of its benefit, incorporated into study designs evaluating subsequent antiviral agents, and eventually, included in guidelines.^27^ Measurement of ALT in the clinical setting is a snapshot of liver inflammation at one moment in time, and may not reflect the dynamic nature of chronic HBV infection with fluctuating levels of ALT elevation. Studies of patients with normal ALT have suggested presence of significant levels of fibrosis and elevated risk of clinical events.^26^, ^28^ Immune responses in the livers of HBV patients, as opposed to the peripheral blood compartment, reveal altered immune function and gene expression, even among patients in the immune control phase (low HBV‐DNA and normal ALT),^29^, ^30^ suggesting that even in the presence of low viral levels and normal ALT, pathogenic effects are present.

HBV‐DNA and ALT are criteria currently included in the assessment and staging of HBV. Given the well‐established association of HBV‐DNA levels with HCC risk, inclusion of ALT may result in the under‐recognition of HBV‐related pathogenic mechanisms already underway. Inclusion of ALT in HBV treatment decisions likely complicates the picture and its role in HBV staging and treatment decisions and warrants reconsideration.

## 3 CLINICAL COURSE OF CHB AMONG PATIENTS IN THE INDETERMINATE PHASE

Recent studies suggest that the indeterminate population comprise a sizeable proportion of the HBV population.^9^, ^10^, ^11^, ^12^, ^13^ A retrospective study involving 3366 adult patients from 5 US clinics and 7 Taiwanese townships over 12.5 years, reported that 39% of enrolled patients were found to be in the indeterminate phase.^9^ The study also reported that these patients were relatively stable, with 53% remaining indeterminate through 10 years of follow‐up.^9^ A report from the North American Hepatitis B Research Network (HBRN) reported 38% of participants were in the indeterminate phase and 64% of them remained indeterminate when using the next available laboratory test.^13^ While most data available on indeterminate phase patients are from Asia, the HBRN data demonstrate that this stage is not limited to Asians, as individuals were classified as indeterminate across racial categories: 16%, 16%, 64% and 3% for Whites, Blacks, Asians and Others, respectively.^13^ Important public health implications arise with such a sizeable proportion of HBV patients falling into the indeterminate stage across multiple studies. With current HBV natural history classifications failing to capture a significant proportion of patients who remain at risk, the natural history and therapeutic framework needs re‐examination. Indeed, rates of HCC have not substantially improved for years, despite the availability of antivirals.^31^, ^32^ Given the effectiveness of NAs in reducing HCC risk, their broader use may have a public health impact.

### 3.1 Clinical outcomes of patients in the indeterminate phase

Clinical data reflect epidemiologic observations connecting viremia with negative outcomes. In a study of 126 HBeAg− patients from India with persistently normal ALT and DNA levels <10^5^ copies/mL, 21% had histologically active liver disease (Histologic Activity Index (HAI) ≥3 and/or fibrosis ≥2).^33^ Similar findings were reported in 193 patients enrolled at a California clinic.^34^ Additionally, HBeAg− patients were more likely to have more advanced histology than HBeAg+ patients (62.7% vs. 47.8%; *p* = .046).^34^ These studies support previous findings showing more adverse hepatic outcomes among patients not meeting treatment criteria at initial presentation when compared to patients who met treatment criteria and were treated.^9^, ^14^, ^26^, ^35^, ^36^, ^37^

A large multi‐national study of 3366 adult non‐cirrhotic HBV patients (38.7% indeterminate) reported a 10‐year cumulative HCC incidence of 4.6% among patients in the indeterminate phase, with a 0.5% incidence reported among inactive patients (*p* < .0001) (adjusted HR = 14.1 (*p* = .03) for developing HCC).^9^ Patients aged ≥45 years who remained in the indeterminate phase had a higher risk of HCC development (HR = 18.4; *p* = .005).^9^ Analytical controls were in place to account for confounding and differences between comparison groups, including large study size and propensity methods.^9^

Other studies have provided additional evidence that patients in the indeterminate phase are at higher risk of HCC development. A Korean study that examined HBeAg− patients with viral levels ≥2000 IU/mL and normal or mildly elevated ALTs (<2× ULN) reported a higher risk of HCC (HR = 1.76; 95% CI: 1.00–3.10; *p* = .05) compared to those treated (ALT ≥2× ULN).^26^ A retrospective multicenter study of 3624 untreated patients examined the occurrence of HCC outside of treatment recommendations and reported 64%, 46% and 33.5% developed HCC outside of The Asia Pacific Association for the Study of the Liver (APASL),^38^ The American Association for the Study of Liver Diseases (AASLD)^39^ and The European Association for the Study of the Liver (EASL)^40^ recommendations, respectively.^41^ A study of a well‐developed HBV cohort in Ethiopia found that WHO guideline criteria failed to detect nearly half of patients in need of therapy when gold standard criteria (including transient elastography) were applied.^42^

### 3.2 Molecular evidence

Hepatocarcinogenic events are believed to occur early over the course of HBV infection. Even among patients in the immune tolerant phase (i.e. HBeAg+, high viral levels with normal ALT), a stage traditionally considered as ‘immunologically tolerant’, and therefore benign, evidence of HBV‐induced immune activation has been described.^43^

During replication, the HBV viral genome can integrate into the host genome at any stage throughout the course of infection,^44^, ^45^ including the earliest stages of acute infection.^44^ Among 26 patients in various stages of HBV, including immune tolerant (*n* = 9), HBeAg+ immune active (*n* = 10), and HBeAg− immune active (*n* = 7),^43^ HBV integrations were observed in all viral genotypes studied as well as in all participants, including those in the immune tolerant phase.^43^ These findings may help explain why approximately one‐third of HCC cases occur in patients without fibrosis.^46^, ^47^ Similarly, RNA sequencing of biopsy samples from the TORCH‐B Study (viremic patients with minimally raised ALT levels 1×‐2× ULN), detected integrations in all patients at baseline and the number of integrations were correlated with markers of viral activity including level of HBV‐DNA, RNA, HBsAg and hepatitis B core‐related antigen (HBcrAg).^48^

An important consequence of integration is increased genomic instability as evidenced by the observation of chromosomal translocation events in 80% of TORCH‐B patients for which sequencing was available.^48^, ^49^ Utilizing long‐read DNA sequencing technology to identify integrated HBV‐DNA sequences flanked by sequences from two different chromosomes, investigators provided experimental evidence for inter‐chromosomal translocations. Previously reported in HBV‐related liver cancer,^50^ translocations may also be present in non‐cancerous samples.^49^ Liver biopsy specimens from a separate study reported 13/42 (31%) samples to contain HBV integrated sequences with evidence for inter‐chromosomal translocations.^51^ Translocations appear to be unique within each biopsy sample, suggesting that they occur randomly, although some samples had evidence of clonal expansion of translocation events.^51^ As translocations can lead to the dysregulation of host gene expression, including oncogenes or tumour suppressors, they highlight another possible hepatocarcinogenic mechanism.

A separate study involving mostly European and African patients, has provided additional evidence for the role of HBV genomic integrations in hepatocarcinogenesis.^52^ Péneau and colleagues reported clonal selection of HBV viral enhancer integrations proximal to cancer‐driver genes as well as evidence for chromosomal rearrangements at integration sites leading to increased expression of cancer‐driver *trans*‐genes.^52^ Importantly, this study also observed a high number of HBV integrations to be associated with viral replication in non‐tumour liver tissues as well as copy number.^52^ The number of integrations was also an independent prognostic factor for HCC.^52^

Integration events appear to be independent of intrahepatic HBV reservoirs in HBeAg− patients, including those with limited HBV reservoirs, and tend to be localized near genes involved in carcinogenesis.^53^ In this study, the investigators divided 84 HBeAg− patients into three groups, based on viral load: HBV‐DNA persistently <2000 IU/mL (Group 1‐ low level viremia); HBV‐DNA 2000–20,000 IU/mL (Group 2‐ moderate level viremia); HBV‐DNA >20,000 IU/mL (Group 3‐ high level viremia) and measured biomarkers including cccDNA, pgRNA, intra‐hepatic HBV reservoir, and integration burden.^53^ Importantly, Groups 1 and 2 reflect viral levels seen in indeterminate HBV patients. While differences between groups were observed in levels of cccDNA, intrahepatic HBV‐DNA, and intrahepatic reservoirs of HBV, a noteworthy finding was the observation that at least 1 HBV‐DNA integration was detected in all patient groups, a finding aligned with the studies described previously.^53^ While the highest frequency of integrations was observed in the high viral level group (Group 3: 55.6%: 10/18), integrations were also present in the low (Group 1: 14.3%: 2/14) and moderate (Group 2: 25%: 2/8) viral groups as well, suggesting that HBV integration occurs in a sizeable proportion of patients in all categories of HBV‐DNA level. Integration events were detected across multiple HBV genotypes and were not associated with patient age, ALT level or Ishak fibrosis stage.^53^ The observation that integrations occur in patients with low viremia with limited intrahepatic HBV reservoirs in multiple viral genotypes and independent of ALT level, underscores the possibility of activated hepatocarcinogenic processes among patients with low viremia and normal ALT.

A retrospective analysis of 287 patients from Spain with a median follow‐up of 8.2 years (range of 5–19 years) reported transition to the inactive carrier phase in 40% of indeterminate patients with none developing advanced fibrosis or cirrhosis.^54^ An Italian study also reported relatively slow progression among 153 patients predominantly infected with genotype‐D.^55^ The relative stability of patients and lack of rapid transition through this stage is supported by several other studies.^9^, ^13^, ^37^ Nevertheless, as described previously, hepatocarcinogenic events are likely present in all stages of HBeAg− infection regardless of HBV genotype,^53^ including those common to non‐Asian countries.^52^, ^53^

Data suggest that hepatocarcinogenesis may be underway early during HBV infection. Evidence for integration events, even in HBeAg− patients with low viremia independent of intrahepatic HBV reservoirs, highlights the existential risk of developing HCC among those with low viral load and normal ALT. Collectively, these data provide evidence for increased HCC risk among patients who do not meet treatment criteria and highlights the question as to whether therapy should be considered to reduce HCC risk.

## 4 IMPACT OF THERAPY ON PATIENTS IN THE INDETERMINATE PHASE

### 4.1 Clinical observations

Evidence of therapeutic impact on patients in the indeterminate phase, both on the molecular and clinical level, come from several studies. Huang and colleagues analysed patients from the REAL‐B Study, a consortium involving 14 centers in the United States, Europe and Asia.^56^ Using AASLD criteria, 2228 patients were classified in the indeterminate phase, of whom 855 patients (405 treated and 450 untreated) met analysis criteria.^56^ The study reported the 5‐, 10‐ and 15‐year cumulative HCC incidence to be 3%, 4% and 9%, respectively, among treated patients, whereas the cumulative HCC incidence was 3%, 15% and 19%, respectively (*p* = .02) in untreated patients.^56^ A consistent pattern for lower 5‐, 10‐ and 15‐year HCC incidence for treated versus untreated patients in subgroup analyses was observed, including patients ≥45 years of age (*p* = .04), male sex (*p* = .03), ALT below the ULN (35 U/L for males and 25 U/L for females) (*p* = .04), HBV‐DNA >1000 IU/mL (*p* = .007) and HBeAg+ (*p* = .0005). As REAL‐B was a retrospective study, propensity score methods were used to adjust for methodological challenges common to retrospective observational analyses.^56^ These findings suggest that treatment may reduce HCC risk among patients in the indeterminate phase.^56^

The TORCH‐B Study was a prospective multicenter, randomized, double‐blind, placebo‐controlled study that investigated the efficacy antiviral therapy on disease progression among predominantly HBeAg− (79% HBeAg−) noncirrhotic patients with HBV‐DNA >2000 IU/mL and minimally elevated ALT (between 1 and 2× ULN).^57^ Among the 160 patients randomized to treatment with tenofovir disoproxil fumarate (TDF) (*n* = 79) or placebo (*n* = 81), 73 individuals in each group completed the study and had adequate liver biopsy sample for histologic evaluation. Fibrosis progression (defined as ≥1 stage increase in Ishak score by biopsy 3 years apart), was significantly lower in the treated group (19/73; 26%) compared to the placebo group (34/73; 47%) (relative risk (RR) = 0.56; 95% CI: 0.35–0.88; *p* = .013).^57^ Although the study was not powered to show a difference in necroinflammation, an overall trend of lower progression of necroinflammation (defined as increase of ≥2 points using the Knodell score) was observed in the treated group, where 5 (7%) progressed compared to 12 (16%) in the placebo group (RR = 0.42; 95% CI: 0.15–1.12; *p* = .084).^57^ Among treated patients, 2/79 (3%) experienced acute hepatitis flares requiring add on entecavir therapy compared to 13/81(16%) in the placebo group (RR = 0.16; 95% CI: 0.04–0.68; *p* = .013).^57^ Given the strong association of increased fibrosis, and particularly cirrhosis, with risk of developing HCC,^47^ these findings reinforce the potential for antiviral therapy to reduce HCC risk.

### 4.2 Molecular observations

In a study of 28 patients who received biopsies at baseline and 1 year, with 7 having an additional one at 10 years, integrations and hepatocyte clonal expansions were detectable in all patients at baseline with a median frequency of 1.01 × 10^9^ integration events per liver.^58^ Following 1 year of NA treatment, the median number of integration events declined by 0.22 logs (*p* = .008), while clone sizes declined by 0.04 logs (*p* = .002).^58^ Among the subset with biopsies at 10 years, an overall decline of 0.93‐logs and 1.02‐logs from baseline for integrations and hepatocyte clone size, respectively,^58^ suggesting a long‐term benefit of NAs as integrations and clone size would not be expected to normalize over this timeframe without treatment. Consistent with reports described previously, integration frequency and hepatocyte clone size were not correlated with HBV viral parameters.^58^

Molecular profiling of paired biopsy samples from TORCH‐B provides additional evidence for the potential benefit of NA therapy. Hsu and colleagues employed total RNA sequencing to quantify the number of distinct transcriptionally active integrations before and after 3 years of therapy in 119 treated patients (*n* = 64) compared to untreated (*n* = 55).^48^ While the number of distinct integrations was comparable between groups at baseline, after 3 years, the number of integration events detected decreased 3.28 and 1.81‐fold in the treatment and placebo groups, respectively (*p* = .037).^48^ Interestingly, the number of distinct viral integrations significantly correlated with serum biomarkers such as HBV‐DNA and HBV RNA levels (*p* < .0003 for all).^48^ These findings are consistent with quantification of integration events using long‐read DNA sequencing in the same population.^48^, ^49^ Collectively, the clinical observations along with molecular data quantifying integrations and immune activation, highlight the potential of antiviral therapy to reduce HCC risk among patients in the indeterminate phase.

## 5 DISCUSSION

In the absence of advanced fibrosis, family history of HCC, or other risk factors for disease progression, current practice guidelines recommend the initiation of antiviral therapy in select stages of HBV where viral levels and ALT levels are elevated. However, a sizeable number of patients cannot be easily classified into one of the stages of HBV and are said to be in an ‘indeterminate phase’ without clear guidance on therapy. Given accumulating evidence that patients in this phase exhibit increased risk for adverse outcomes such as HCC, molecular data that hepatocarcinogenic mechanisms may already be underway, and data that antiviral therapy could help reduce the risk of HCC, it is important from both patient and public health standpoints, that patients in this phase are recognized by the natural history framework and providers given appropriate guidance on therapy. The proposed WHO guidelines should move in this direction with simplified treatment criteria that may potentially reduce the number of patients in the indeterminate phase.^7^

Data highlighting increased HCC risk among indeterminate patients have largely been retrospective in nature. Recent studies, like the one conducted by Huang and colleagues,^9^ utilized statistical approaches such as propensity score methods, to adjust for potential biases present in observational research and their findings are supported by data from earlier studies which demonstrated a relationship between of viral levels and increased HCC risk. The REVEAL HBV Study demonstrated a clear relationship between increasing HBV‐DNA levels with increasing risk of cirrhosis,^19^ and HCC.^18^ In both cases, the risk was higher at HBV‐DNA levels ≥10,000 copies/mL (~2000 IU/mL), and were independent of HBeAg status and ALT level.^18^, ^19^ While higher risk was seen with HBV‐DNA ≥10,000 copies/mL, lower viral levels (<10,000 copies/mL) were still associated with risk, albeit at much lower levels.^18^, ^19^ Collectively, these findings highlight the role of HBV‐DNA, even in the absence of elevated ALT.

Studies showing HBV‐DNA integrations and chromosomal translocations provide a picture of ongoing hepatocarcinogenic mechanisms among patients in the indeterminate phase. Importantly, evidence has shown that NAs appear to reduce the burden of integrations and translocations in liver biopsies, features of genomic instability and characteristic of carcinogenesis.^51^, ^52^, ^58^ Even in an early landmark study demonstrating the effect of lamivudine therapy on reducing HCC incidence, the beneficial effects were seen irrespective of ALT levels.^59^ Taken as a whole, the data continue to underscore the importance of viremia in contributing to negative HBV outcomes.

Natural history studies have reenforced the relationship between HCC and low levels of viremia in the setting of normal ALT in HBeAg− patients.^18^ Retrospective analysis of a large multi‐center and multi‐national cohort demonstrated a clear benefit of antiviral therapy for reducing HCC risk.^60^ By utilizing fibrosis as a surrogate endpoint over a defined 3‐year timeframe, TORCH‐B provided clinical evidence that antiviral therapy lowers the risk of progression in liver fibrosis among those with viremia and minimally elevated ALT.^57^ Collectively, these data provide a picture of the potential long‐term benefit of therapy on patients who are in the indeterminate phase for reducing HCC risk. Moreover, molecular studies demonstrate that putative drivers of hepatocarcinogenesis such as viral integrations, chromosomal translocations and immune activation are present in these patients and are reduced following antiviral treatment.

Many guidelines fail to classify a significant portion of HBV patients in the indeterminate phase. With accumulating data suggesting that these patients are at risk of hepatic morbidities that can be ameliorated by NAs in a cost‐effective manner,^61^ it can be argued that any residual risk of HCC should not be tolerated among patients in the indeterminate phase.

## AUTHOR CONTRIBUTION

LJY and CF conceptualized the manuscript. LJY developed the initial draft of the manuscript. All authors (LYM, RJM, CR, YCH, LJY and CF) contributed to the writing and editing of the manuscript. All authors have read and agreed to the final draft of the manuscript.

## CONFLICT OF INTEREST STATEMENT

**Lung‐Yi Mak**: consults for Gilead Sciences and has received lecture fees from AbbVie. **Robert J. Wong**: Research grants (to his institution) from Gilead Sciences, Exact Sciences, Theratechnologies and Durect Corporation. Has served as consultant (without compensation) for Gilead Sciences. **Christian Ramers**: Consults for, advises, is on the speakers' bureau and has received grants from Gilead; he consults for, advises, is on the speakers' bureau for AbbVie. **Leland J. Yee** and **Carrie Frenette**: employees of, and own stock in, Gilead Sciences. **Yao‐Chun Hsu**: Yao‐Chun Hsu has served as an advisory committee member for Gilead Sciences and Sysmex, has spoken for AbbVie, Bristol‐Myers Squibb, Gilead Sciences, Merck Sharp & Dohme and Roche, and has received research grants from Gilead Sciences, all within the past 5 years.

## Data Availability

The information contained in this review was compiled from publicly available sources and does not contain new data.
